# A Comparative Study on the Shaping Ability and Cleaning Efficiency of Two Different Single-File Systems, Reciprocating Wave One Versus Continuous Rotation F360, Evaluated by Scanning Electron Microscope: An In Vitro Study

**DOI:** 10.7759/cureus.37503

**Published:** 2023-04-12

**Authors:** Arunkumar Samudrala, Chandrakanth Majeti, Kommineni Harika Chowdary, Lakshmi Bhavani Potru, Anusha Yaragani, Yata Prashanth Kumar, Gagandeep K Sidhu, Navneet S Kathuria

**Affiliations:** 1 Conservative Dentistry, Maharaja Ganga Singh Dental College and Research Centre, Sriganganagar, IND; 2 Department of Conservative Dentistry and Endodontics, SB Patil Dental College and Hospital, Bidar, IND; 3 Conservative Dentistry and Endodontics, Tirumala Institute of Dental Sciences, Nizamabad, Nizamabad, IND; 4 Conservative Dentistry and Endodontics, Sibar Institute of Dental Sciences, Guntur, IND; 5 Orthodontics and Dentofacial Orthopaedics, Konaseema Institute of Medical Sciences (KIMS) Dental College, Amalapuram, IND; 6 Conservative Dentistry and Endodontics, Government Dental College and Hospital, Hyderabad, IND; 7 Oral Pathology and Microbiology, Maharaja Ganga Singh Dental College and Research Centre, Sriganganagar, IND

**Keywords:** waveone file, smear layer, root canal treatment, f360 file, biomechanical preparation

## Abstract

Background*:* In endodontics, the three processes of biomechanical preparation, disinfection, and obturation are each essential. The electron microprobe and scanning electron microscope (SEM) allowed for the detection and identification of the smear layer and debris. The purpose of the current investigation was to use a scanning electron microscope to evaluate the relative merits of two single-file systems, the reciprocating WaveOne and the continuous motion F360 files, in the cleaning and contouring of root canals in removed teeth.

Materials and method*:* The 50 central maxillary permanent teeth Data was gathered from the Oral and Maxillofacial Surgery Division at Sri Ganganagar's Maharaja Ganga Singh Dental College and Research Centre for a number of reasons. Group A followed the manufacturer's guidelines for using the WaveOne instrument, whereas Group B utilised the F360. Reciprocating motion WaveOne system (group A) and continuous motion F360 system (group B) root canals were scored at three levels: coronal third, middle third, and apical third (group B). SPSS version 22 was used for the data analysis. The data were examined using the chi-square test and the one-way analysis of variance.

Results*: *A greater quantity of smear layer was found in the apical third, whereas better results were achieved in the coronal and middle thirds. When compared to the F360 file system, the WaveOne file system is subpar when it comes to clearing the canal of debris. While both groups showed a large amount of debris in the apical third, outcomes were somewhat better in the coronal and middle thirds.

Conclusion*: *The WaveOne and F360 file systems were both more effective in removing trash from the coronal and middle thirds of the disc than they were from the apical thirds. In comparison to the continuous motion F360 file system, WaveOne files demonstrated a statistically significant reduction in the amount of debris cleared from root canals in all three root zone thirds (coronal, middle, and apical). The reciprocating action of the WaveOne file system, in contrast to the continuous motion of the F360 file system, resulted in more extensive cleaning of the root canal smear layer in the coronal and middle thirds and less thorough cleaning in the apical thirds of the canal.

## Introduction

Endodontic therapy has seen explosive growth over the last several decades as an increasing number of patients have desired to preserve their natural teeth. Endodontic operations have progressed to the point that the majority of patients are pleased with the outcomes of their treatments [1,2]. The shaping of the canals inside the tooth is one of the aspects of root canal treatment that is considered to be among the most important. It consists of removing potentially pathogenic material from the root canal system, inclusive of necrotic pulp tissue, dentine debris, and microbes [1]. It is dependent on this that subsequent treatments, like chemical disinfection and root canal obturation [2], are successful.

Each of the three procedures of biomechanical preparation, cleaning, and obturation is necessary for performing endodontic treatment. Because the mineralized tissues of the dentine are broken rather than shredded or divided, the process of cutting dentine, whether by hand or with rotary machinery, results in a significant amount of debris. This makes up the majority of the smear layer and consists of very minute particles of mineralised collagen matrix. It is a layer that was created during the smearing process. It is possible that there is a layer that is 2-5 µm thick and goes on for a few micrometres into the dentinal tubules [3]. Dentine chips and bits of necrotic or live pulp tissue that have been affixed to the wall of the root canal are also examples of debris [4]. This material has a chance of being compacted along the surface of the root canal walls. This is a possibility. When this occurs, it becomes more challenging to eliminate germs from the canal system in an efficient manner, and it also raises the danger of bacterial contamination, which may result in an unfavourable outcome of therapy. The electron microprobe and scanning electron microscope (SEM) were both helpful in locating and identifying the smear layer and the debris.

Although several varieties of rotary systems have been in use for several decades, the dental industry has only recently welcomed two new single-file systems, one of which operates in a "reciprocating motion" and the other in a "continuous motion." Both of these new single-file systems operate in a manner that is described as "continuous motion" [5,6].

A reciprocating motion is a motion that occurs when an instrument rotates in one direction and then reverses direction before completing a full rotating cycle. This motion is referred to as a "back and forth" motion [5].

The formation of the canals often makes use of both continuous and reciprocating procedures. Both advantages and disadvantages are associated with making use of a continuous rotating movement as opposed to a reciprocating motion. When employing Ni-Ti files, which spin continuously, the user is able to have an experience that is both more exact and more pleasant. This is an extra benefit. These worries have been alleviated to a significant degree because of developments in file design, Ni-Ti alloy, and sequential glide path management (GPM). The glide path enlarges the root canal to create a smooth tunnel from the orifice to its physiological terminus before using shaping instruments of greater dimensions [6].

In addition, there is a paucity of information on the effect that continuous motion or reciprocation motion has on the process of removing the smear layer from the dentinal wall. Practitioners could profit from this information if they use more care when designing canals in areas where the weaker component of the file is intended to work. The current research was conceived with the intention of analysing and contrasting the degree of success achieved by two distinct single-file systems, namely the reciprocating WaveOne and the continuous motion F360 files, in terms of their capacity for root canal cleaning and shaping.

## Materials and methods

The 50 central maxillary permanent teeth were obtained from the Oral and Maxillofacial Surgery Division of Sri Ganganagar's Maharaja Ganga Singh Dental College and Research Centre. This in vitro study was conducted at the Maharaja Ganga Singh Dental College and Research Centre in Sri Ganganagar, Rajasthan. After receiving approval from the institute's ethical committee, it was carried out in the Department of Conservative Dentistry and Endodontics, in collaboration with the Department of Oral Pathology.

After being stored in normal saline and rinsed with tap water to remove any remaining blood or tissue, the teeth were ultrasonically cleaned and placed in the refrigerator (at 40 °C) prior to instrumentation to facilitate their fracture (longitudinal section) for scanning electron microscopy examination. This was done in order to facilitate the examination of the teeth using a scanning electron microscope. An endo access bur was used in order to make an opening, and the working length was determined by inserting a size 10 K file into the apical foramen until the minor diameter was attained. In all, there were 50 teeth, and these teeth were divided into halves so that each section included 25 teeth. A solution comprising 5.25% sodium hypochlorite, 17% EDTA, and saline was alternatively used to irrigate two sets of cells. Saline was used as the control.

In group A (WaveOne, rotational/movemental symmetry), the WaveOne instrument was operated in accordance with the instructions provided in the handbook. Using a K file of size 10, made of stainless steel, the optimum working length was found. The chamber was then filled with 3% sodium hypochlorite so that the process of sculpting the coronal expansion file could begin. After that, we use a WaveOne main file with a size of 0.25 and a taper of 0.06, rotating it in a reciprocating motion at 1.8 N-cm torque and 300 rpm speed with a Marathon A-Class endomotor. This allows us to ream out the normal space that is found in a root canal. In group B, the F360 instrument was used exactly as described, which was continuous rotation/motion using the F360. Using a K file of size 10, made of stainless steel, the optimum working length was determined. Before beginning the process of moulding the component by using a coronal expansion file, the chamber was first loaded with 3% sodium hypochlorite. An F360 single file with a 0.06 taper was used to continually travel through the root canal region while a Marathon A-Class endomotor with a control torque of 1.8 N-cm and a speed of 300 rpm was used. This was done in order to clean the area.

From the very beginning of the procedure, both the amount and the frequency of the irrigation were subjected to regulation (the preparation phase). When cleaning and flushing each canal, 2 ml of sodium hypochlorite with a concentration of 5.25%, 2 ml of EDTA with a concentration of 17%, and 2 ml of saline were used. The irrigant was administered with the use of a needle syringe and a no. 25 gauge needle, and the needle was inserted into the canal to a depth that was as far as it could go without becoming stuck. After flushing the canal for one minute with an EDTA solution that was 17%, it is then thoroughly washed with 2 ml of EDTA, which was 5.25%, and 2 ml of saline to eliminate any residual irrigants. Following the irrigation and instrumentation of the canals, the channels were then dried using paper points. The technique for irrigation that was devised by Zmener et al. [7] was adhered to throughout this experiment.

In the end, absorbent paper points and irrigants were used to dry and clean the root canals. Irrigants were utilised to clean the root canals. Before being prepared for scanning electron microscopy, the crown was removed by slicing it off with a diamond disc wheel, and the roots were sectioned in half longitudinally by slicing a diamond disc in half (at 1000×).

**Figure 1:**
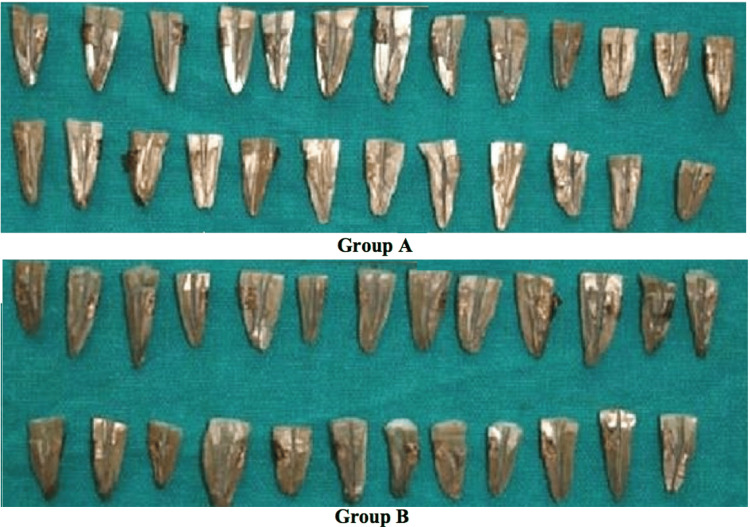
Samples coated with gold palladium

The samples (Figure 1) from groups A and B were each cut into three sections - coronal, middle, and apical - before being analysed using a scanning microscope with a magnification setting of ×1000. This was done in order to identify the presence and degree of debris and smear layers. In the end, the scoring technique that was provided by Hulsmann et al. [8] was the one that was chosen, and the criteria for the scores are described in Tables 1-2.

**Table 1:** Debris scoring criteria [8]

Score	Debris scoring criteria
Score 1	Clean canal wall, only very little debris particles
Score 2	Few conglomerations of debris present
Score 3	Many conglomerations <50% of the canal wall covered
Score 4	>50% of the canal wall covered with debris
Score 5	Complete or nearly complete covering of the canal wall by debris

**Table 2:** Smear layer scoring criteria [8]

Score	Smear layer scoring criteria
Score 1	No smear layer, orifice of dentinal tubuli patent
Score 2	Small amounts of smear layer some dentinal tubuli patent open
Score 3	Homogenous smear layer along almost the entire canal wall, only very few dentinal tubuli open
Score 4	The entire root canal wall is covered with homogenous smear layer, no open dential tubuli
Score 5	A thick homogenous smear layer covering the entire root canal wall

**Figure 2:**
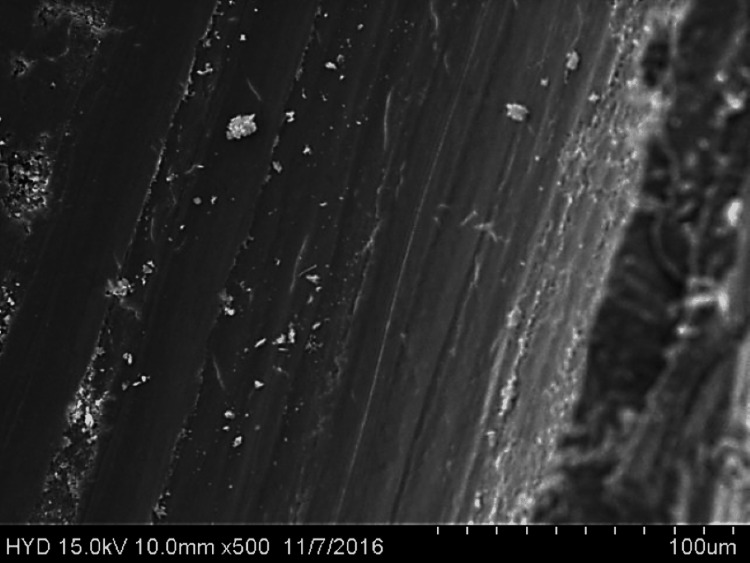
Photomicrograph of root canal shaping with reciprocating WaveOne motion (coronal third)

**Figure 3:**
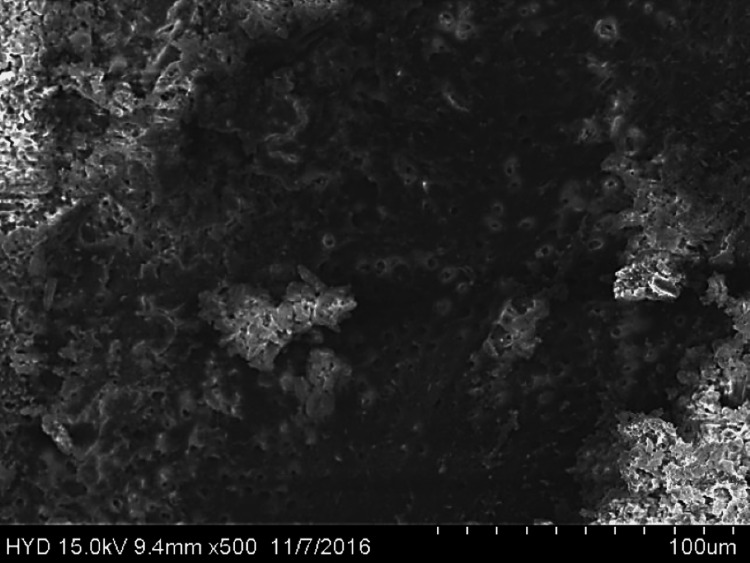
Photomicrograph of root canal shaping with reciprocating WaveOne motion (middle third)

**Figure 4:**
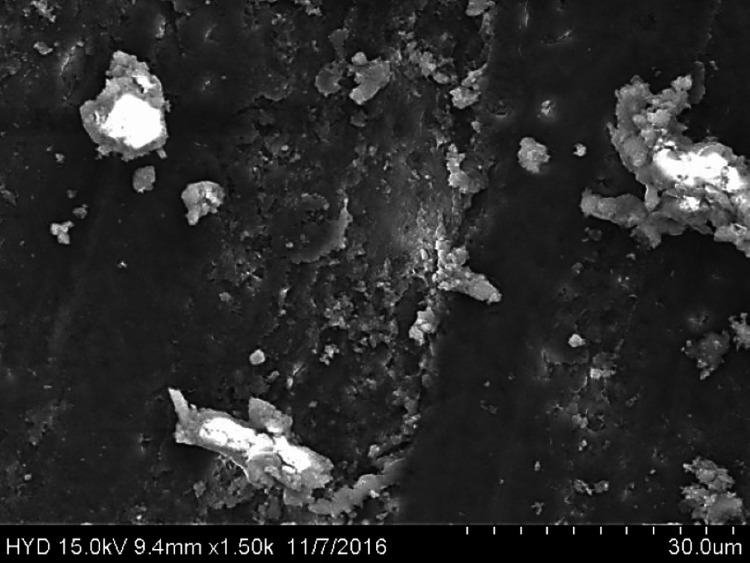
Photomicrograph of root canal shaping with reciprocating WaveOne motion (apical third)

**Figure 5:**
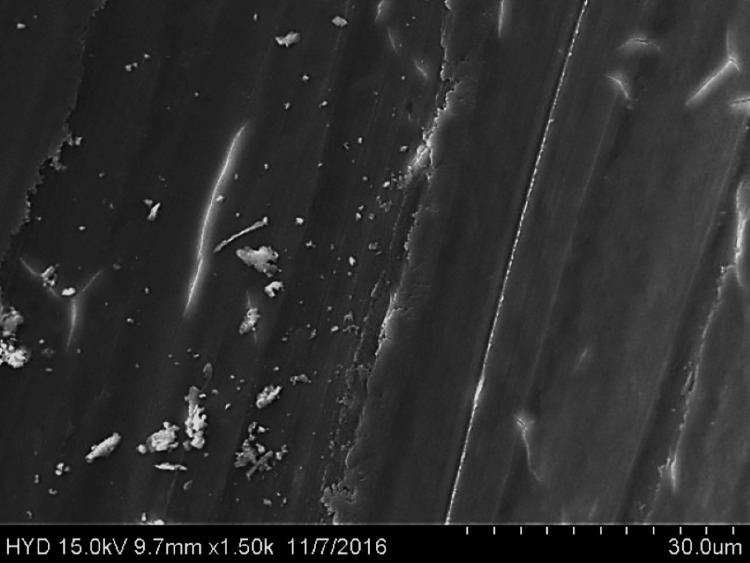
Photomicrograph of root canal shaping with continuous motion F360 (coronal third)

**Figure 6:**
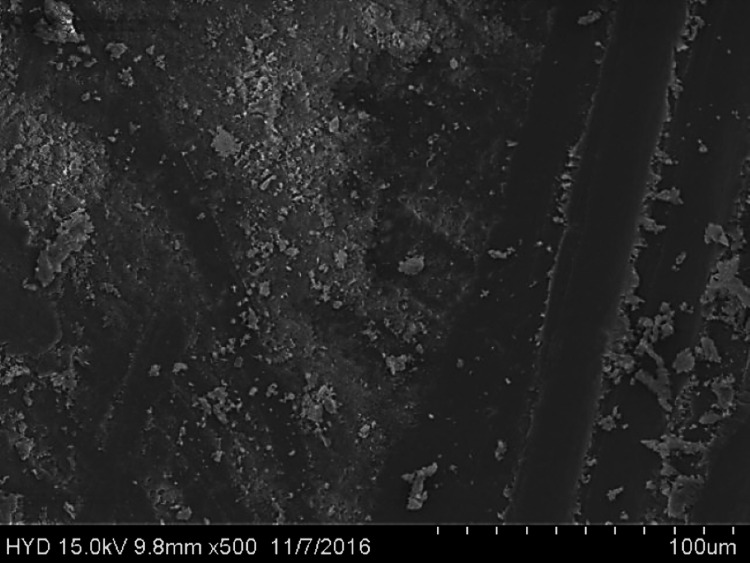
Photomicrograph of root canal shaping with continuous motion F360 (coronal third)

**Figure 7:**
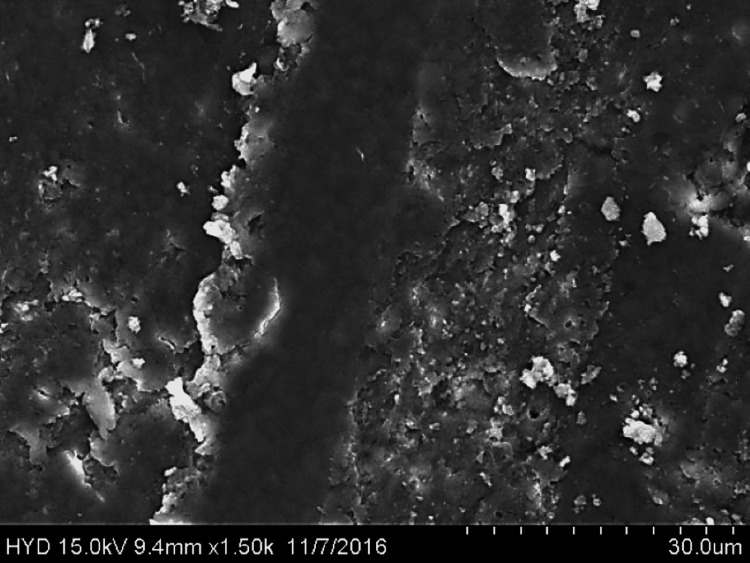
Photomicrograph of root canal shaping with continuous motion F360 (apical third)

Fifty specimens were examined by scanning electron microscopy; 25 were assigned to group A (Figures 2-4), while the remaining 25 were assigned to group B (Figures 5-7).

Three fields of each specimen were examined at ×1000 magnification, for a total of 75 fields per group (group A: 3 × 25 = 75; group B: 3 × 25 = 75). In this paper, we compare groups A (the reciprocating motion WaveOne system) and B (the continuous motion F360 system) based on the scoring criteria assessed at three distinct levels of root canals. SPSS version 22 (IBM Corp., Armonk, NY) was used to analyse the data using chi-square and one-way ANOVA.

## Results

On the basis of the results of the present study for the reciprocating WaveOne system (group A) and continuous F360 system (group B), Table 3 reveals that for the reciprocating WaveOne system (group A) in the coronal third, it is found that 68% of specimens had scores of 1, 20% of specimens had scores of 2, and 12% of specimens were found with a homogeneous layer of smear layer covering the root canal with few dentinal tubules open. In the middle third, 60% of specimens were found with a little amount or without a smear layer (score 1), 28% of specimens were found with a score 2, and 12% were found with a score 3. In the apical third, the smear was abundant, and only 52% of samples were found with a score 1, 32% of samples with a score 2, 8% of samples with a score 3, and 8% of samples of canals were completely covered with a smear layer.

**Table 3:** Comparison of smear layer removal score **Statistically significant (p<0.001).

Smear layer scoring	Score description	Coronal		Middle		Apical	
		Group A	Group B	Group A	Group B	Group A	Group B
		n=25	n=25	n=25	n=25	n=25	n=25
1	No smear layer, dentinal tubules open	17	15	15	14	13	17
		68%	60%	60%	56%	52%	68%
2	Small amount of smear layer, some dentinal tubules open	5	5	7	6	8	6
		20%	20%	28%	24%	32%	24%
3	Homogenous smear layer covering root canal few dentinal tubules open	3	5	3	5	2	2
		12%	20%	12%	20%	8%	8%
4	Complete root wall covered by smear layer, no dentinal tubules present	0	0	0	2	2	0
		0%	0%	0%	8%	8%	0%
5	Heavy non-homogenous smear layer covering the entire root	0	0		0		0
		0%	0%	0%	0%	0%	0%
Significance		P<0.001**		P<0.001**		P<0.001**	

For the continuous motion F360 system (group B) in the coronal third, it is found, as shown in Table 3, that 60% of specimens showed little or no smear layer (score 1), 20% of samples were found with a score 2, and 12% of samples with a score 3. For the middle third, 56% of cases had little or no smear layer, 24% had a small amount of smear and some dentinal tubules were open, and 12% had a homogeneous smear layer covering the root canal space. In the apical third, 68% of the specimens were found with little or no smear layer, 24% of samples were found with a small amount of smear layer, and 8% of samples had a homogeneous smear layer present on the canal walls.

On the basis of the results of the study for the reciprocating WaveOne system (group A) and continuous motion F360 system (group B) (dentin chips, pulp remnants, and particles loosely attached to the canal wall), the debris scores for the reciprocating WaveOne system (group B) are as revealed in Table 4. In the coronal third, 64% of samples were found with score 1, 24% were found with score 2, and in the middle third, 68% of specimens were found with score 1, 28% were found with score 2, and 12% were found with many agglomerulations of debris covering less than 50% of the root canal wall. In the apical third, debris was abundant, and only 40% of samples were found with a clear root canal wall and canals with only a few small debris particles; 28% of samples were found with score 2, 20% of samples were found with many agglomerulations of debris covering less than 50% of the root canal wall (score 3), and 12% specimens of canals were found with more than 50% of the root canal wall covered with debris. For the continuous F360 system (group B), the debris scores used for the criteria are as demonstrated in Table 4. In the coronal third, 60% of samples were found with score 1, 24% were found with score 2, and 16% were found with score 3. In the middle third, 60% of specimens were found with score 1, 36% were found with score 2, and 4% were found with many agglomerulations of debris covering less than 50% of the root canal wall. In the apical third, 52% of samples were found with a clear root canal wall, with only a few small debris particles, 36% were found with score 2, 8% of samples were found with many agglomerulations of debris covering less than 50% of the root canal wall (score 3), and 4% specimens of canals were found with more than 50% of the root canal wall covered with debris (score 4).

**Table 4:** Comparison of debris removal score between two groups *Statistically significant (p<0.05).

Smear layer scoring	Coronal		Middle			Apical		
	Group A	Group B	Group A	Group B	Group A		Group B	
	n=25	n=25	n=25	n=25	n=25		n=25	
Clear root canal wall, only a few small debris particles	14	15	15	15	10		13	
	56%	60%	60%	60%	40%		52%	
Few Small agglomerluations of debris present	9	6	7	9	7		9	
	36%	24%	28%	36%	28%		36%	
Many agglomerulations of debris covering less than 50% of root canal wall	2	4	3	1	5		2	
	8%	16%	12%	4%	20%		8%	
More than 50% of the root canal wall covered	0	0	0	0	3		1	
	0%	0%	0%	0%	12%		4%	
Complete or nearly complete root canal wall with debris	0	0	0	0	0		0	
	0%	0%	0%	0%	0%		0%	
Significance	0.016*		0.114			0.247		

**Figure 8:**
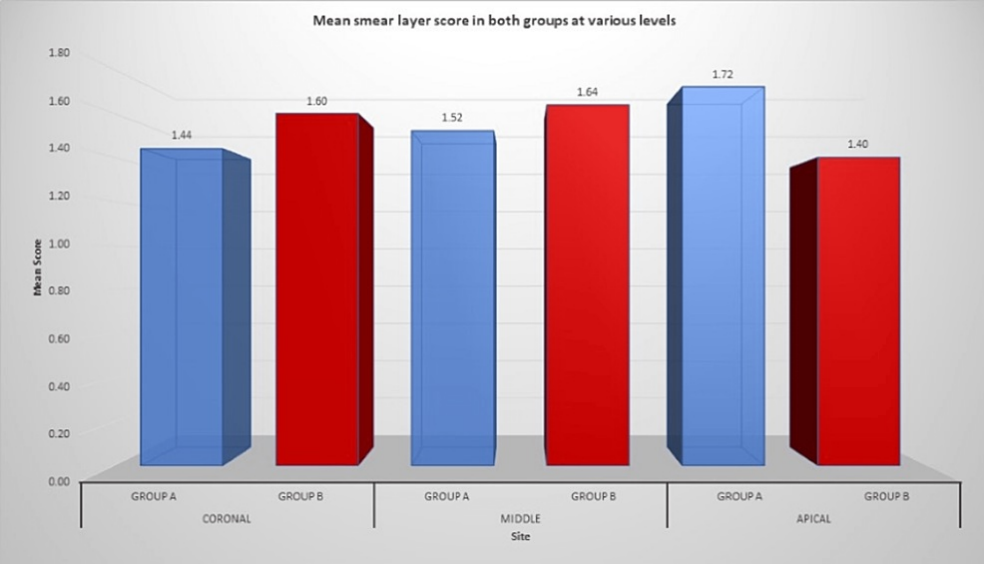
Mean smear layer score in both groups at different levels

Figure 8 shows the mean smear layer score in both groups at different levels.

**Figure 9:**
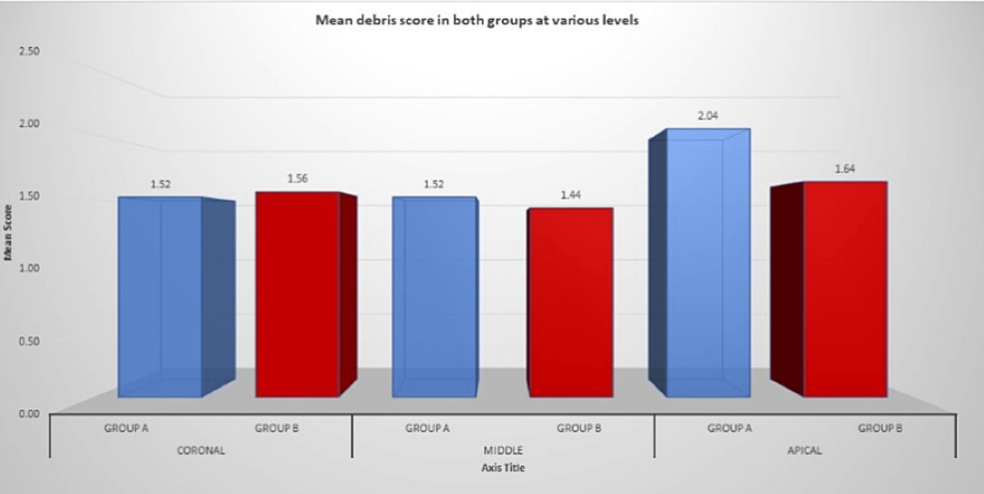
Mean debris score in both groups at different levels

Figure 9 shows the mean debris score in both groups at different levels.

No significant differences (p<0.01) were found between the two used systems, the single file reciprocating motion system and the continuous motion F360 system (Table 5). However, concerning the elimination of the smear layer, better results were obtained in the coronal and middle thirds, compared to the apical third, where a high amount of smear layer was observed. After analysis of the obtained results, it can be concluded that WaveOne presented a higher degree of removal of the smear layer of root canals in the coronal and middle thirds and less in the apical thirds than the F360 file system (p<0.01).

**Table 5:** Overall comparison of smear layer score (one way ANOVA) NS: not significant (P>0.05). Inference: there is no statistically significant differences present in the smear layer score at various levels in either group A or group B.

		Sum of squares	df	Mean square	F	Sig.
Group A	Between groups	1.040	2	0.520	0.824	0.443 NS
	Within groups	45.440	72	0.631		
	Total	46.480	74			
Group B	Between groups	0.827	2	0.413	0.713	0.494 NS
	Within groups	41.760	72	0.580		
	Total	42.587	74			

However, concerning the elimination of debris from the canal, the WaveOne file system was unable to clear the debris properly from the canal than the F360 file system. Somehow, better results were obtained in the coronal and middle thirds, compared to the apical third, where a high amount of debris was observed in both groups. The analysis of the results concluded that WaveOne presented a lower degree of removal of debris from the root canals in the coronal, middle, and apical thirds with a statistical difference when compared with the continuous motion F360 file system (p<0.05) (Table 6).

**Table 6:** Overall comparison of debris score (one way ANOVA) *Statistically significant (p<0.05), NS: not significant (P>0.05). Inference: there is statistically significant difference present in the debris score of group A at various locations, no difference in debris score in group B at various levels.

		Sum of squares	df	Mean square	F	Sig.
Group A	Between groups	4.507	2	2.253	3.282	0.043*
	Within groups	49.440	72	0.687		
	Total	53.947	74			
Group B	Between groups	0.507	2	0.253	0.479	0.621 NS
	Within groups	38.080	72	0.529		
	Total	38.587	74			

## Discussion

The objective of biomechanical instrumentation is to thoroughly clean the root canal region of any diseased pulp tissue, including remnants of the pulpal tissue, debris, and a smear layer [8]. The thickness of the smear layer, which is a thin coating that remains on the root canal wall after instrumentation, ranges anywhere from 1 to 2 µm. A smear layer could not be found in the areas that were not instrumented. The smear layer is composed of residual irrigants, pieces of dentine, germs, and the remains of the live or necrotic pulp tissue. Dentine fragments also contribute to this layer [9].

The purpose of the present study was to compare the cleaning efficacy of root canals using different rotary single file systems: WaveOne (Dentsply Maillefer, Ballaigues, Switzerland), which works in reciprocating motion, and F360 (Komet Brasseler GmbH & Co., Lemgo, Germany), which works in continuous motion. Yared in 2008 introduced a new approach using a single NiTi instrument that works in reciprocating movement (single-file root canal preparation) with only 1 ProTaper F2 (Dentsply Maillefer, Ballaigues, Switzerland), which was used in a reciprocating motion [5]. The reduction in the number of files has led to the assumption that the apical extrusion of debris and the release of neuropeptide could be decreased, therefore reducing the prevalence of symptomatic apical periodontitis [10-12].

The first single-file and single-use files launched in the market were made for use in a reciprocating motion. Although single-file reciprocating systems have been shown to offer advantages over multi-file rotary systems, by using reciprocating motion, the risk of cyclic fatigue fracture is reduced because it is presumed that the counterclockwise rotation of reciprocating motion diminishes the torsional stress exerted on the file during the active canal shaping procedure [13].

The WaveOne (Dentsply Maillefer) NiTi single-file system is relatively new and is designed to be used with a dedicated reciprocating motion. Todays last single file systems are developed to be used in continuous rotatary motion and not for reciprocating motion; the F360 (Komet Brasseler GmbH & Co., Lemgo, Germany) file system belongs to them. The WaveOne system, which is a single-file reciprocating system with unequal clockwise/counterclockwise motion, has been claimed to be four times safer and almost three times faster than when using multiple rotary files to achieve the same final shape [10,14]. The WaveOne system is a single-file reciprocating system with asymmetrical clockwise/counterclockwise motion [14]. F360, which is also a single-file system, was first introduced to work in continuous motion with 4% and 6% taper. These files have a modified S-shaped cross-section, which helps more in clearing the debris. The pre-sterilised, single-use F360 files are designed to prevent cross-contamination, eliminate the need to clean, disinfect, and sterilise the instruments, and reduce the risk of fracture due to cyclic fatigue [15].

Both of these tools were used in this research in a manner that adhered to the recommendations provided by the manufacturers of each tool. All of the canal spaces inside the root samples were appropriately prepared by adhering to all of the operation sequences and adjusting the instrument operating parameters. In addition to analysing the conclusions drawn from the preparation of the canal space, using a scanning electron microscope at a greater magnification (1000×), a comparison of the cleaning performance of two instrumentation approaches was performed by numerical evaluation of the debris and smear layer in the canal's coronal, middle, and apical regions (SEM).

In this present study, both tested instruments were used in accordance with the manufacturer's instructions. All protocol sequences and instruments' operative settings were respected, the canal spaces of all root samples were prepared accordingly, and the results of the outcome of the preparation of the canal space were evaluated. The cleaning efficacy of the two instrumentation methods was examined on the basis of separate numerical evaluations of the coronal, middle, and apical portions of the canal debris and smear layer at higher (1000×) magnification under SEM. Both reciprocating motion WaveOne and continuous motion F360 file systems effectively removed the smear layer in the coronal and middle thirds but not the apical third in the present study. In comparison to the WaveOne file system, which works in reciprocating motion and effectively removes the smear layer in the coronal and middle thirds but leaves more smear layers in the apical thirds, the F360 file system works in continuous rotation. F360, which works in continuous motion, showed more effective cleanliness in the apical third of the canals. In a similar study, conducted by Kockac et al. [16], no difference was found between the two instruments in terms of the presence of smear layers, pulpal debris, and inorganic debris, and the scores were significantly higher in the reciprocating WaveOne file than the continuous motion OneShape file. However, in another study by Poggio et al. [17], it was concluded that reciprocating instruments seemed to be less effective in promoting the cleanliness of root canal walls and in removing the smear layer from dentine. It was found that reciprocating instruments leave a higher quantity of smear layer, and due to this fact, dentinal tubules are not completely opened. Another study carried out by Dagna et al. [18] found that the F6 Skytaper seems to be more effective than the F360 in the middle third, probably due to the increased taper. However, each backward motion of the reciprocating file might provide the opportunity for debris to build up in protrusions and isthmus areas [17]. In addition, the reciprocating motion of the file may not allow the blade to cut into the dentine as cleanly, resulting in a burnishing-type effect and pushing debris into recesses and isthmuses [19].

Amaral et al. [20] conducted research on the removal of the smear layer in canals that had been shaped using reciprocating and rotary systems, such as Reciproc, WaveOne, or Mtwo systems. The researchers found that there were no statistically significant differences between the three methods, particularly in the middle and coronal thirds of the canal. It was discovered that the thickness of the smear layer increased as one moved closer to the apex of the structure. Suparna et al. [21] evaluated the cleaning effectiveness of two different rotary file systems using a scanning electron microscope. These systems were the ProTaper NEXT and the WaveOne. The researchers found that the rotating systems WaveOne and ProTaper NEXT cleaned the coronal and middle thirds of the root canal much more successfully than the apical thirds of the canal. The single-file system in reversible motion offered by WaveOne beat Protaper NEXT in terms of performance.

When compared side by side, the outcomes of a continuous rotational motion and those of a reciprocating motion reveal that the latter generates a preparation that is more well-balanced. The steady rotating movement has very little effect on the canal's inner curvature; nevertheless, it does have the effect of enlarging the canal's exterior, especially in the third that is closest to the apex. Because of this, it should come as no surprise that the reciprocating movement is responsible for higher cleaning efficiency [22]. The reciprocating movement, which helps in root canal cleaning, has a number of advantages, including a reduction in torsional and flexural stresses, an improvement in canal centering ability, and a reduction in the amount of taper lock that occurs inside the root canal [23,24].

The removal of dentine with WaveOne is shown to be mild and inadequately comprehensive in the SEM pictures. In spite of its modest surface activity, it generates a thick, less soluble smear layer that EDTA is unable to remove. This layer cannot be removed by washing. The propensity of this file system is to produce a smear layer that is "well packed" and not one that should be desired. Because of the rotating motion of NiTi instruments, it is probable that they disseminate and compress microscopic dentine particles and shavings along dentine walls. These particles and shavings are then partially dissolved by EDTA and removed coronally via flute gaps. The F360, which has a cross-section in the shape of a "double S" and two cutting blades, is the ideal instrument for the removal of debris. It also produces SEM pictures that are often free of the smear layer and display the majority of dentinal tubules in unconstrained and completely open conditions.

In comparison to the F360 file system, which has a larger triangular cross-section, the WaveOne file system may not have sufficient space for debris to be transported, which in turn reduces the cutting ability and cleaning efficiency of the WaveOne file system. Because it is required to remove cut dentin pieces in order to minimise clogging of the cutting blades, the cross-sectional design of mechanical instruments also has an influence on how well they are cleaned [25,26]. Bürklein et al. [27] compared the cutting effectiveness of the Reciproc and the WaveOne instruments and found that the S-shaped cross-section of the Reciproc instrument was more effective than the triangular cross-section of the WaveOne instrument. They concluded that the S-shaped cross-section of the Reciproc instrument was superior to the triangular cross-section of the WaveOne instrument. In addition to this, they discovered that the configuration of the cross-section was more essential than the kind of motion that was employed for cutting and cleaning.

It is also claimed that when instrumentation is performed with files of greater taper, a pseudo sense of snug fit is created because of the binding of the flutes in the coronal portion, whereas the apical portion may lie uninstrumented [28]. Also, irrigants may not penetrate the apical thirds, which have narrower diameters than the coronal and middle thirds. All these factors may be responsible for the better cleaning of the coronal and middle thirds when compared to the apical thirds [29]. Hence, the meticulous way of using different instruments plays a vital role in the proper biomechanical preparation of the root canal as well as being required to obtain a better and cleaner root canal to obtain asepsis.

In the present study, only upper maxillary central incisors were considered because, in straight access canals, the removal of debris is better appreciated than in curved canals [30], although further studies on curved canals are recommended.

The limitation of the present study is that WaveOne seems to produce a "well-packed" smear layer, which is not a desirable property of this file system. Furthermore, it is an in vitro study, and further in vivo studies comparing the efficacy of both systems are recommended.

## Conclusions

The present study concludes that both the WaveOne and F360 file systems effectively cleared debris in the coronal and middle thirds but not in the apical thirds. However, in comparison, the WaveOne file system presented a lower degree of removal of debris from the root canals in the coronal, middle, and apical thirds with a statistical difference when compared with the continuous motion F360 file system. Furthermore, there were no statistically significant differences in the removal of the smear layer at various levels in both the WaveOne file system and the F360 file system. However, in comparison, the WaveOne file system, which works in a reciprocating motion, presented a higher degree of removal of the smear layer of root canals in the coronal and middle thirds and less in the apical thirds than the F360 file system, which works in continuous motion.
